# Pollution Characteristics, Spatial Distribution, and Evaluation of Heavy Metal(loid)s in Farmland Soils in a Typical Mountainous Hilly Area in China

**DOI:** 10.3390/foods12030681

**Published:** 2023-02-03

**Authors:** Guohui Shen, Xin Ru, Yanting Gu, Wei Liu, Kunzhen Wang, Baiyi Li, Yanzhi Guo, Juan Han

**Affiliations:** 1 Institute of Food and Nutrition Development, Ministry of Agriculture and Rural Affairs, Beijing 100081, China; 2 North China Geological Prospecting and Ecological Resources Monitoring Center (Hebei), Chengde 067000, China; 3 Department of Research Management, Chinese Academy of Agricultural Sciences, Beijing 100081, China

**Keywords:** ecological risk, enrichment factor, geo-accumulation index, geostatistical analysis, Nemerow index, principal component analysis

## Abstract

Heavy metal(loid)s pollution in farmland soil is not only a serious environmental but also a human health-related issue. Accurate understanding and evaluation of heavy metal pollution levels in the soil are very important for sustainable agricultural development and food safety. Mountainous and hilly areas have the dual functions of industrial development and agricultural production, and the farmland soil in these areas is more susceptible to heavy metal pollution. In this study, the single factor index, Nemerow index, geo-accumulation index, enrichment factor index, and potential ecological risk indices, which are mainly used to assess the contamination and risk of heavy metals in farmland soils. The sources of heavy metals in agricultural soils of the study area were analyzed using correlation analysis and principal component analysis. Finally, geostatistical methods were used to map the heavy metal contamination of farmland soils. An average concentration of all heavy metals (except As) in farmland soils of the study area exceeded the corresponding background values, as indicated by the obtained results. The results of the principal component analysis showed that the heavy metal sources in the soils of the study area can be classified into two groups. The five pollutant index methods all showed the most serious Hg pollution in the study area. The integrated pollutant mapping results showed that the risk of heavy metal pollution in the study area was mostly moderate, except for the western and central parts of the region. This study enhances understanding of the pollution levers of heavy metals in Yiyuan farmland soils, and also can facilitate the monitoring of heavy metal contaminants at the primary stage of the food chain and assess the risk of the presence of heavy metal contaminants in food, thus improving the health of the residents.

## 1. Introduction

Soil is not only indispensable to human beings and the basis for their survival but also an important medium for all kinds of life activities on earth. Nowadays, as the rapid economic and social development, environmental quality of soil is declining under the influence of industrial pollution and chemical fertilizers and pesticides [1,2]. Heavy metals have a wide range of biological toxicity, non-degradability, and accumulation in living organisms, easy to produce enrichment in the soil; human activities generate large amounts of heavy metal emissions, which can easily lead to secondary pollution [3,4,5]. Soil heavy metal pollution not only directly affects the quality of agricultural products through the biosphere cycle but also can be indirectly absorbed by the human body, thereby threatening human health [6,7,8]. As an indispensable hub of material and energy exchange in agricultural ecosystems, it is of great significance to explore the characteristics, ecological risk status, and pollution sources of heavy metal pollution in soil for the prevention and control of heavy metal pollution. Based on data from China’s State Environmental Protection Administration (SEPA), china has faced serious soil heavy metal contamination (CSC 2012). Approximately 10 million hectares of arable land in China are contaminated, and about 12 million tons of grains are contaminated with heavy metals in the soil every year [9]. The treatment of heavy metal pollution on farmland has become a priority for pollution control in China [10].

Research on soil heavy metal contamination began mainly in industrial cities; in recent years, research has been expanding to include roads, parks, and agricultural soils [11,12,13,14]. The accumulation of heavy metals in farmland soils and various indicators for ecological risk assessment have caught the interest of many academics worldwide [15]. Among them, the pollution index [16], the geo-accumulation index [17,18], Nemerow integrated pollution index [19], the enrichment factor [20,21,22], and the potential ecological risk index [23,24] were widely used to evaluate the heavy metal pollution characteristics and ecological risk of agricultural soils. Most studies have shown that complex assessment models make pollutant assessment results more accurate and comprehensive [25,26].

Mountainous and hilly areas have the dual functions of industrial development and agricultural production. However, because they are far from human settlements, it is easier to ignore the risk of heavy metal pollution in the soil. Therefore, it was a priority to study the heavy metal pollution of farmland soil in these areas. In this study, 0–20 cm of agricultural soil in Yiyuan County, Shandong Province, a typical mountainous and hilly area, was used as the research object. This study uses a combination method of pollution index, Nemerow integrated pollution index, geo-accumulation index, geostatistical analysis, enrichment factor, potential ecological risk index, and principal component analysis, aiming to analyze the pollution level, spatial distribution, and ecological risk of cultivated soils in Yiyuan County, Shandong Province, China, as well as to quantitatively analyze the potential sources of heavy metals. The main objectives of this research are to determine the concentrations and distributions of heavy metals (As, Cd, Co, Cr, Cu, Hg, Mn, Ni, Pb, and Zn) in soils around farmland in mountainous and hilly areas, identify the sources of heavy metals in soil using principal component analysis, realize mapping soil heavy metal pollution distribution based on geostatistics spatial analysis technology, and to assess the pollution levels and potential ecological risks to human beings of the heavy metals in the farmland soils.

## 2. Materials and Methods

### 2.1. Study Area

**Figure 1:**
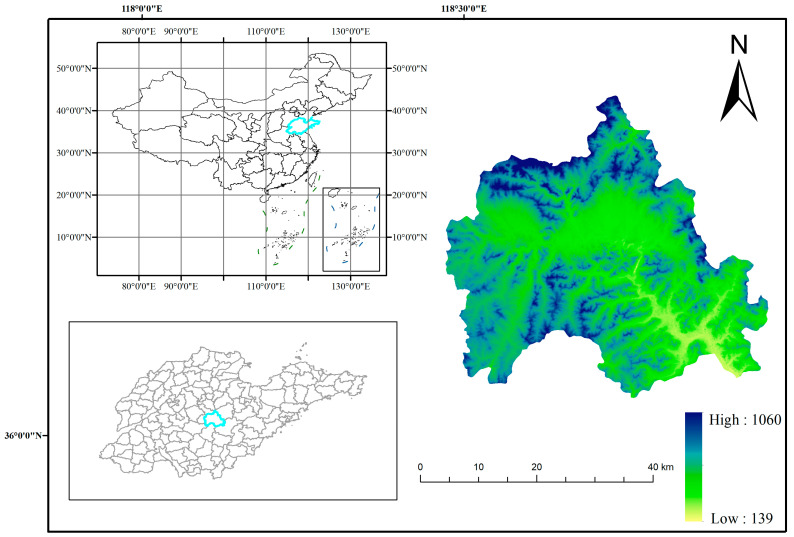
Overview of the study area.

**Figure 2:**
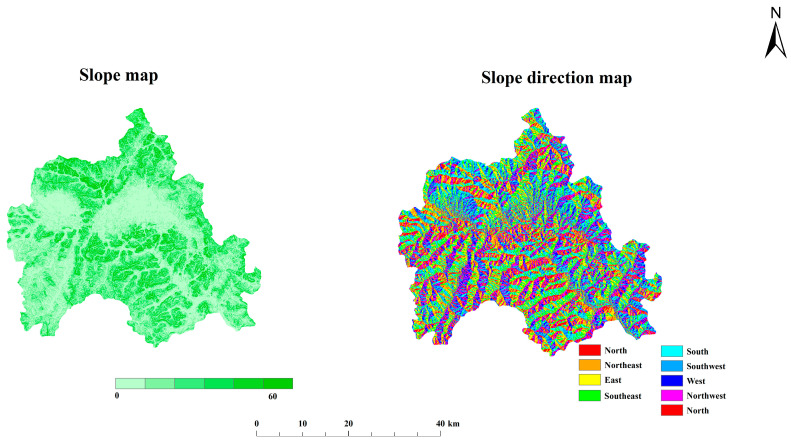
Slope orientation map of the study area.

Yiyuan County, which belongs to Zibo City in central Shandong Province, is located at latitude 35°55′~36°23′ N and longitude 117°48′~118°31′ E (Figure 1). It is the best latitudinal zone for apple production in the world. It belongs to the warm temperate monsoon zone and has a continental climate in which the average annual temperature is 12.5 °C while the average annual precipitation is 712 mm. The undulating mountains of Yiyuan County, with an average elevation of 400 m above sea level (Figure 2), are the highest average elevation in Shandong Province. The geomorphological type is mainly mountainous hills, with the total terrain being high in the northwest and low in the southeast. The substrate is the ancient metamorphic system of the Taishan Group; soil-forming parent material is mainly limestone, granite, and slope deposits. The light ratio is the highest in China, the forest coverage is 48%, and the soil is loess, sandy and loamy, rich in mineral nutrients such as potassium and calcium. The fruit industry is the “backbone” of the local agricultural economy, and 70% of farmers’ income comes from the fruit industry economy. The industrial enterprises in the study area are located in the central county, while some coal mining, chemical and ore processing enterprises are scattered in the region.

The blue part shows the topographic elevation distribution of the study area.

### 2.2. Soil Sampling

**Figure 3:**
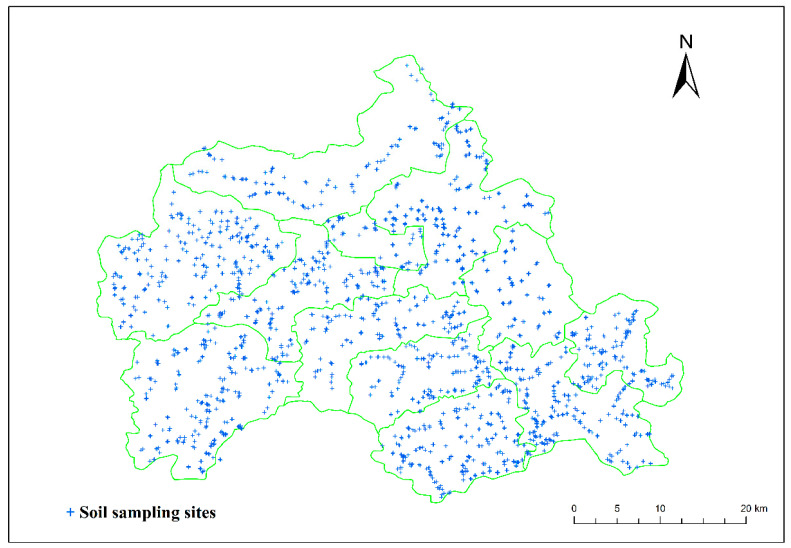
Distribution of farmland soil sampling points.

A soil field survey in the investigated area was conducted to accomplish the current research objectives. In the present study, the method of sample collection used was that described in “NY/T 395-2012” (MAPRC, 2012). The sampling points were eventually adjusted according to village distribution and land use type, and the soil profile locations were registered in the field using a Garmin GPS and then plotted on a map (Figure 3). Based on the soil morpho-logical variation, 3290 (0–20 cm) representative soil samples were taken from the various horizons of the investigated profiles. Laboratory soil samples were collected from five replicates at each sampling site and manually mixed to form a composite sample. Samples that had been air-dried were crushed, sieved through a 2 mm sieve, and then stored in polyethylene containers for various examinations. Excluding latitude, longitude, and some elements missing data, finally, 3060 sample data were obtained.

### 2.3. Chemical Analysis

Ten heavy metals, namely, As, Cd, Co, Cr, Cu, Hg, Mn, Ni, Pb, and Zn, were evaluated in this study. The soil samples were digested as per the procedure detailed in “HJ/T 166-2004” (CEPA, 2004), and after soil dissolving by the HNO_3_-HCl-HF-HClO_4_ method, for the determination of 10 heavy metal items. Among them, Cr, Cu, Ni, Pb, and Zn were determined by plasma optical emission spectrometry (ICP-OES), Cd, Co, and Mn were determined by graphite furnace atomic absorption spectrophotometry (GF-AAS), and AS and Hg were determined by atomic fluorescence spectrometry (HG-AFS). The analytical methods were tested for accuracy and precision using national-level soil standards (GBW series), while stringent sample quality monitoring was conducted through proportional sampling and exception checks, with results meeting monitoring requirements [27].

The specific testing details of each element are as follows.

#### 2.3.1. Cr, Cu, Ni, Pb, and Zn

The samples were decomposed with nitric acid, perchloric acid and hydrofluoric acid, then dissolved in aqua regia (1 + 1), fixed into 25 mL PTFE test tubes, fixed and shaken well, and left overnight. The characteristic spectral intensities of the components to be measured in the test solution were measured by inductively coupled plasma emission spectrometer at the specified wavelengths, and the effects of the matrix were corrected to calculate the amount of the components to be measured in the specimen.

#### 2.3.2. Cd, Co, and Mn

Weigh 0.2500 g of soil sample in a Teflon crucible, moisten with appropriate amount of water, place the crucible on a temperature-controlled electric heating plate in a fume hood, add 5 mL of hydrochloric acid and 5 mL of nitric acid, cover the crucible, heat to 11 °C for 1 h, remove and cool slightly, then add 4 mL of hydrofluoric acid and 1.5 mL of perchloric acid, heat to 200 °C, continue to heat for 1.5 h, remove the crucible cover and heat again. 1.5 h, raise the temperature to 380 °C, wait until the white smoke is exhausted, add 3 mL of nitric acid solution with a volume ratio of 1:1 to dissolve the residue by warming, rinse the inner wall of the crucible with water, take off and cool, transfer to a 50 mL volumetric flask and fix the volume with water to the scale.

#### 2.3.3. AS and Hg

Determination of As: The specimen should be less than 74 μm in particle size and placed in a ground-mouth glass vial for backup. Weigh 0.2500 g of the sample, accurate to 0.0001 g, place the sample in a 50 mL stoppered test tube, add a small amount of water to wet it, add 10 mL (1 + 1) aqua regia, cover the stopper and shake well. Place in boiling water for 1 h, shake twice in between, remove and cool, fix the volume to 25 mL, shake well and leave overnight. Dispense 5 mL of the clear solution in a small beaker, add 2.5 mL of 1 g/L iron salt solution and 2.5 mL of thiourea-ascorbic acid mixed solution, shake well, leave for 30 min, and then use potassium borohydride solution as reducing agent to determine As by atomic fluorescence spectrometer.

Determination of Hg: The particle size of the specimen should be less than 74 μm and placed in a ground-mouth glass vial for backup. Weigh 0.2500 g of the sample, accurate to 0.0001 g, place the sample in a 50 mL stoppered test tube, add a small amount of water to moisten, add 10 mL (1 + 1) aqua regia, cover the stopper and shake well. Place in boiling water and keep for 1 h, shake twice in between, remove and cool, fix the volume to 25 mL, shake well and leave overnight. Add 2 mLSnC_l2_ solution in hydride generator, then dispense 2 mL solution in atomic fluorescence spectrometer to determine Hg.

#### 2.3.4. pH and SOM

The pH was determined in a 1:2.5 soil-to-water suspension at 25 °C by a pH meter [28]. The soil organic matter (SOM) was measured using the Walkley–Black method [29].

The detection limits of each element are shown in Table 1.

### 2.4. Evaluation Methods for Heavy Metals

This study assessed the contamination levels of ten heavy metals in agricultural soil samples by five methods: the pollution index ($P_{i}$), the geo-accumulation index ($I_{geoi}$), the enrichment factor ($EF_{i}$), the Nemerow index ($P_{n}$), and the environmental risk index (RI). A comparison and discussion of the results of these five estimation methods are also presented.

#### 2.4.1. Single Factor Index [30]

The Single factor index was calculated by using Equation (1):

(1) $$P_{i}=\frac{C_{i}}{S_{i}}$$

$P_{i}$ is a single contamination of heavy metal i, where $C_{i}$ stands for the element i concentration in the soil sample, and $S_{i}$ stands for the background value of element i.

#### 2.4.2. Nemerow Index [30]

The Nemerow composite index was calculated by using Equation (2):

(2) $$P_{n}=\sqrt{\frac{P_{imax}^{2}+P_{iavg}^{2}}{2}}$$

$P_{n}$ represents the Nemerow integrated pollution index of soil heavy metal i, $P_{imax}$ represents the maximum value of soil heavy metal pollutant index, and $P_{iavg}$ represents the average value of soil heavy metal pollutant index.

#### 2.4.3. Geo-Accumulation Index [31]

The geo-accumulation index was calculated by using Equation (3):

(3) $$I_{geoi}=log_{2}(\frac{1}{k}*P_{i})$$

$I_{geoi}$ represents the geo-accumulation index of heavy metal i, $P_{i}$ is a single contamination of heavy metal i, and k is a background matrix correction factor that includes possible variations of the background values due to lithogenic effects, and is systematically taken as 1.5.

#### 2.4.4. Enrichment Factor Index [32]

The enrichment factor index was calculated by using Equation (4):

(4) $$EF_{i}=\frac{{\left(C_{i}/C_{s}\right)}_{sample}}{{\left(B_{i}/B_{s}\right)}_{background}}$$

$EF_{i}$ is the enrichment factor index of heavy metal i, where $C_{i}$ and $C_{s}$ are the same as above, $B_{i}$ is the concentration of the reference metal, and $B_{s}$ is the background value of the reference elements.

#### 2.4.5. Potential Ecological Risk Index [33]

The potential ecological risk Index was calculated by using Equations (5)–(7):

(5) $$RI=\sum E_{r}^{i}$$

(6) $$E_{r}^{i}=T_{r}^{i}\times C_{r}^{i}$$

(7) $$C_{r}^{i}=\frac{T_{fact}^{i}}{C_{n}^{i}}\;$$

where $C_{r}^{i}$ is the pollution index of heavy metal i, $T_{fact}^{i}$ and $C_{n}^{i}$ are the actual and standard values of heavy metal i, respectively; $E_{r}^{i}$ is the potential ecological risk index of heavy metal i, $T_{r}^{i}$ is the Toxicity index; RI is the integrated potential ecological risk index.

In this paper, all elemental background values are selected from Shandong Province [34]. The toxicity index of Mn, Zn, Cr, Cu, Pb, Ni, Co, As, Cd and Hg were 1, 1, 2, 5, 5, 5, 5, 10, 30 and 40, respectively. The enrichment factor reference element was chosen Sc. Table S1 shows the relevant pollution index determination criteria.

### 2.5. Statistics Analysis

In this study, SPSS 23.0 (Chicago, IL, USA) was used to perform statistical analysis, correlation analysis and principal component analysis for ten heavy metals. The geostatistics software of GS+9.0 (USA) was used to complete the variogram fitting and determine the relevant parameters. ArcGIS 10.2 (ESRI Redland, CA, USA) was used to perform kriging operations. Graphics production using R 4.02 (Wellington, New Zealand) and Origin 2021 (Northtampton, MA, USA). The raw data were identified as outliers using 3 sigmas and replaced using the average value.

## 3. Results

### 3.1. Concentrations of Heavy Metals

According to summary statistics (Table 2), the content of As, Cd, Co, Cr, Cu, Hg, Mn, Ni, Pb and Zn varied in the range of 3.06~17.73, 0.03~0.27, 7.29~20.76, 20.57~115.80, 3.48~83.48, 0.01~0.21, 330.40~996.50, 12.53~49.69, 9.45~54.12, 35.14~135.30, 0.24~7.34, 4.54~8.79. According to Wilding [35], the soil properties coefficient of variation (CV) is classified into three categories: low variation (CV < 15%), moderate variation (15% < CV < 35%), and highly variable (CV > 35%). The coefficients of variation of Cd and Hg all reached 35%, which was highly varied. The heavy metal of As, Co, Cr, Cu, Mn, Ni, Pb, and Zn were moderate variations. We also tested the pH and SOM of local farmland. The result indicated that the mean pH value of the soil was 7.36, which was alkaline. The study area of farmland SOM content in China belongs to the medium level. The mean levels of Cd, Co, Cr, Cu, Hg, Mn, Ni, Pb, and Zn were above the background values of 1.27, 1.20, 1.01, 1.68, 2.78, 1.20, 1.10, 1.39, and 1.36, correspondingly.

### 3.2. The Relationships among Soil Properties

**Figure 4:**
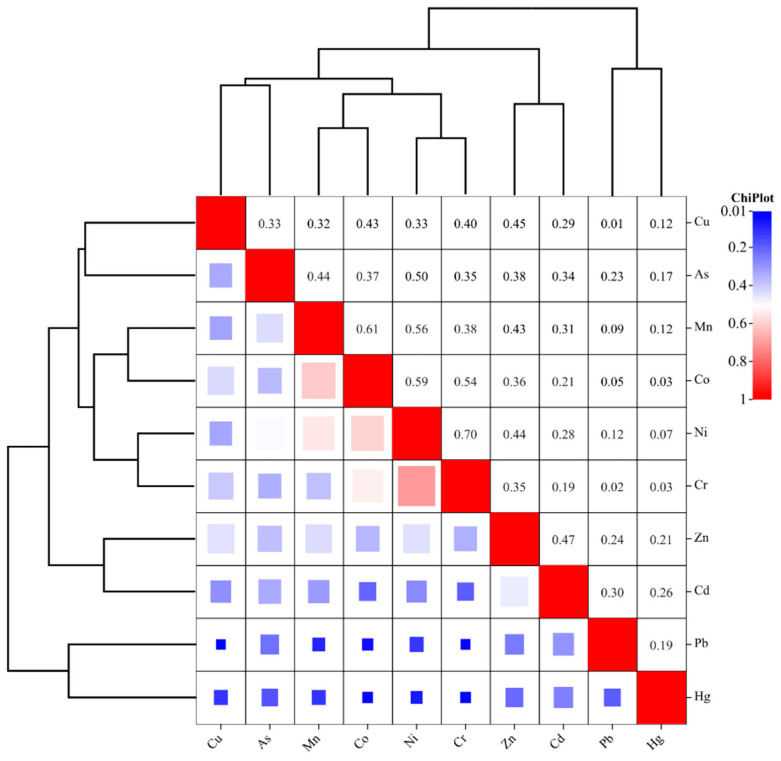
Correlation analysis among soil heavy metals.

Soil heavy metal correlation analysis can deduce if the source of heavy metals are the similar. When there is a significant positive correlation between elements, it indicates that there are similar sources among elements; if there is a significant negative correlation between the elements, it indicates that there are differences in the sources of the elements, and there may be some antagonistic effect. The results indicated that there was a significant positive correlation between all soil heavy metals except Hg and Co, Cr, Pb, Cr, and Cu (Figure 4). This indicated that most of the heavy metals in the study area share the same source. We also found that most of the heavy metals were significantly and positively correlated with soil pH, excluding Hg and Pb. Meanwhile, As (r = 0.062, *p* < 0.01), Cr (r = 0.039, *p* < 0.05), Ni (r = 0.072, *p* < 0.01) and Zn (r = 0.046, *p* < 0.05) were significantly and positively correlated with soil organic matter (Table 3). All heavy metal elements are positively correlated, indicating that heavy metal pollution of farmland soils in the region may have the same source.

### 3.3. Principal Component Analysis

Principal component analysis (PCA) has been widely used in soil quality evaluation because of its outstanding features in optimally integrating and simplifying high-dimensional variable systems, objectively determining the weights of each variable, and avoiding supervisory arbitrariness. Principal component analysis can reveal the structure of soil contaminant data and the intrinsic association of contaminants and describe the main processes of soil contaminant distribution [36,37]. The results of Kaiser–Meyer–Olkin (KMO = 0.822, *p* < 0.001) and Bartlett’s sphericity tests conferred that the concentrations of HMs in farmland soils were suitable for PCA. The results of the rotated principal component loading matrix showed that the first two principal components with eigenvalues greater than 1 and cumulative contribution of 54.28% could explain most of the information of the 10 heavy metal content data, so the first two principal components could be selected (Table 4). The table shows the results of the rotated component matrix; the first principal component (PC1) was mainly composed of Ni, Co, Cr, and Mn; these four metals represent the main information of PC1. The Factor matrix of Cd, Pb, and Hg on the second principal component (PC2) was greater than 0.6, and the common information of these three heavy metal elements was reflected on the second principal component.

### 3.4. Spatial Distribution of Heavy Metals

**Figure 5:**
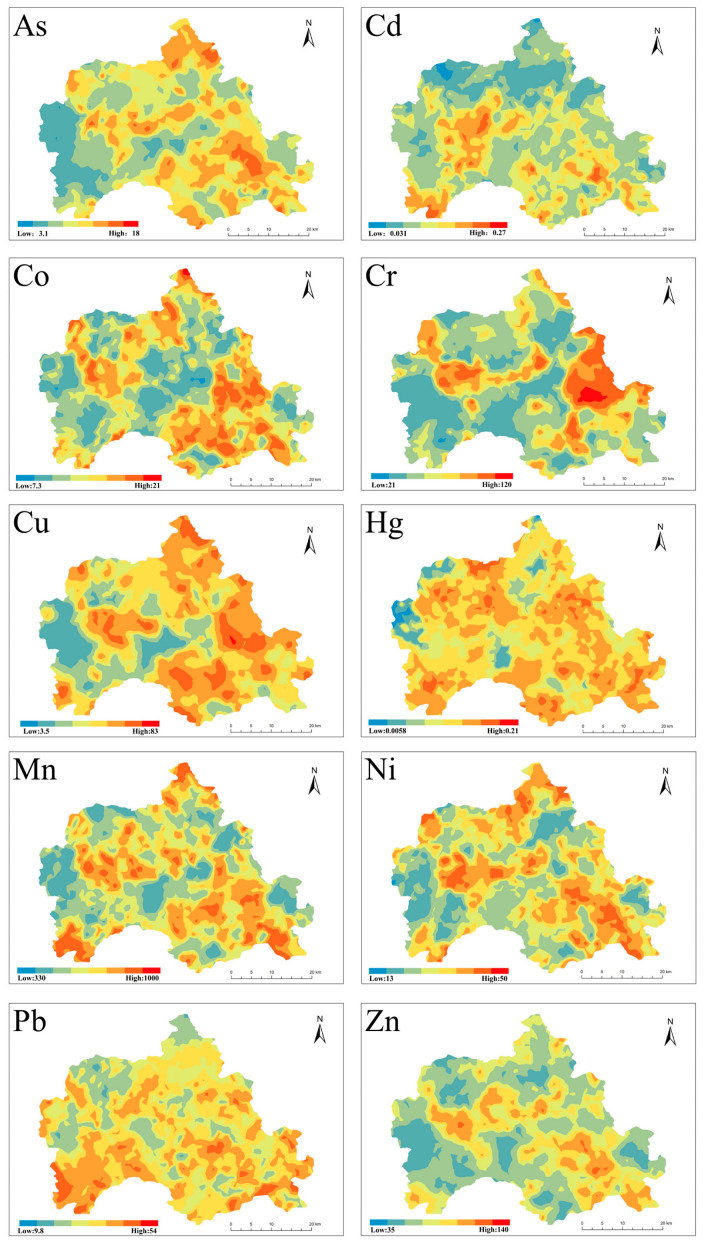
Soil heavy metals distribution map.

Geostatistical analysis can effectively identify areas of high pollutant concentrations, and ordinary Kriging is the most effective tool [38,39]. The results of the distribution map of heavy metals in farmland showed that the spatial distribution of high-value areas of these ten heavy metal concentrations was similar (Figure 5). Most of the high-value areas for heavy metals were distributed in plain areas with relatively scattered. Yiyuan County is a typical mountainous and hilly area where human activities are mainly concentrated in the plain area, residential areas, and factories are also located. Thus, the high-value areas of heavy metal possibly related to human activity factors. From the overall distribution of heavy metals, the east is more enriched in heavy metals than the west, with more high-value areas.

### 3.5. Evaluation of Heavy Metal Pollution in Farmland Soil

Heavy metals can be absorbed by plants from the soil, which can accumulate in the plants and enter the human body through human diet, causing certain hazards to human health. Therefore, a reasonable assessment of the current status of soil heavy metal contamination is essential for crop cultivation and human health. This section focuses on a series of methods to assess the current status of soil heavy metal contamination in the study area.

#### 3.5.1. Single Factor Index Method

**Figure 6:**
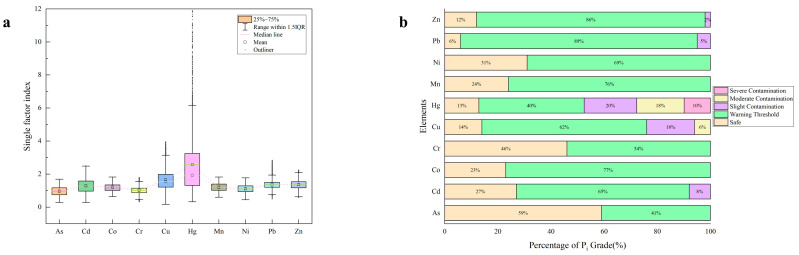
(**a**) Single factor index (P_i_) of HMs in Farmland soil and (**b**) the percentage of P_i_ grade (%).

The average single factor index values of As, Cd, Co, Cr, Cu, Hg, Mn, Ni, Pb and Zn in samples were 0.96, 1.30, 1.20, 1.01, 1.68, 2.57, 1.20, 1.10, 1.39, and 1.36, respectively (Figure 6a). This indicated that all heavy metals except Hg (within the warning threshold) fall within the safe range. As shown in Figure 6a, in the case of As, 59% of single factor index values were safe, while 41% of single factor index values were in the range of 1–2, which was the warning threshold. For Co, Cr, Mn and Ni, the safe percentages were 23%, 46%, 24% and 31%, respectively. The percentages of warning threshold were 77%, 54%, 76 and 69%. The single factor index values of Cd in 23% of soil samples were safe, while single factor index values of 65% and 7% were warning thresholds and slight contamination (Figure 6b). The evaluation results of the single-factor index method showed that the contamination levels of the 10 HMs in the soil samples were largely variable, with the accumulation of these HMs being almost entirely influenced by human activities.

#### 3.5.2. Nemerow Composite Index

The result of the Nemerow composite index showed that As, Cd, Co, Cr, Mn, Ni, and Zn were slight contamination, Pb was moderate contamination, and Cu and Hg were severe contamination. The values of the Nemerow composite index decreased in the order of Hg > Cu > Pb > Cd > Zn > Mn > Co > Ni > Cr > As. Among them, the contamination of Hg and Cu was the most serious, reaching 8.61 and 3.05, respectively (Table 5).

#### 3.5.3. Geo-Accumulation Index

**Figure 7:**
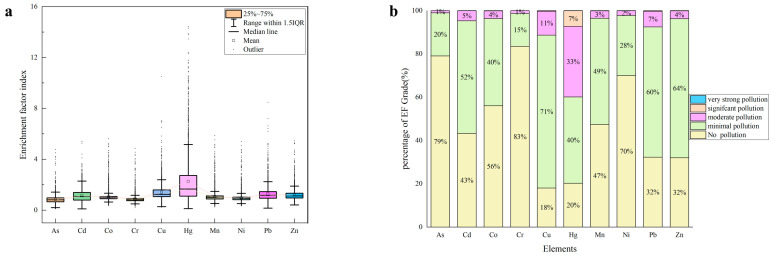
(**a**) geo-accumulation index (*I_geoi_*) of HMs in Farmland soil and (**b**) the percentage of geo-accumulation index grade (%).

The geo-accumulation index (*I_geo_*), often called the Muller index, this index can not only indicate the characteristics of natural variation in the distribution of heavy metals, but also distinguish the impact of human activities on the environment. It is an important parameter to distinguish the impact of anthropogenic activities [40,41]. The geo-accumulation index (*I_geo_*) values of heavy metals in the farmland soil samples were calculated based on the geochemical background values of soils in the Shandong Province of China. The values of *I_geo_* ranged from −2.36 to 0.17 for As, −2.41 to 0.74 for Cd, −1.23 to 0.28 for Co, −2.22 to 0.27 for Cr, −3.18 to 1.41 for Cu, −2.23 to 2.99 for Hg, −1.31 to 0.28 for Mn, −1.75 to 0.24 for Ni, −1.59 to 0.93 for Pb, and −1.36 to 0.59 for Zn. The average *I_geo_* values of As, Cd, Co, Cr, Cu, Hg, Mn, Ni, Pb and Zn were −0.73, −0.31, −0.36, −0.62, 0.04, 0.45, −0.36, −0.49, −0.14 and −0.17, respectively (Figure 7a). This indicated that As, Cd, Co, Cr, Mn, Ni, Pb, and Zn were unpolluted, and Cu and Hg were unpolluted to moderately polluted. In addition, the *I_geo_* values of As, Cd, Co, Cr, Mn, Ni, Pb, and Zn were <1 in all samples, and only a small number of *I_geo_* values for Cu and Hg were >1 (Figure 7b), while 7% of the soil samples in Hg were moderate to heavily polluted.

#### 3.5.4. Enrichment Factor Index

**Figure 8:**
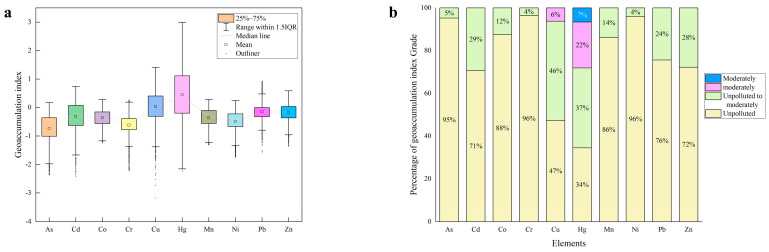
(**a**) Enrichment factor index (EF) of HMs in Farmland soil and (**b**) the percentage of Enrichment factor index grade (%).

Enrichment factors (EF) is commonly used to assess the level of enrichment of heavy metals in soils and to determine their sources [42]. The reference elements are usually more stable elements such as Mn, Fe, Al, Sc, Ti, etc. [43,44]. In this study, Sc was selected to be the reference element in the setting studied here, and the EF values of As, Cd, Co, Cr, Cu, Hg, Mn, Ni, Pb, and Zn elements were calculated. The results showed that the EF values ranged from 0.11 to 4.79 for As, 0.11 to 5.40 for Cd, 0.36 to 5.61 for Co, 0.20 to 4.87 for Cr, 0.24 to 10.51 for Cu, 0.12 to 31.88 for Hg, 0.26 to 5.87 for Mn, 0.33 to 5.40 for Ni, 0.6 to 8.48 for Pb, and 0.41 to 5.45 for Zn, respectively. The average values of As, Cd, Co, Cr, Cu, Hg, Mn, Ni, Pb, and Zn were 0.83, 1.14, 1.06, 0.88, 1.42, 2.27, 1.06, 0.97, 1.28, and 1.20, respectively (Figure 8a). According to the classification criteria of EF, the farmland soil samples in the study area were not contaminated with As, Cr and Ni, while Cd, Co, Cu, Mn, Pb and Zn had low levels of contamination, as well as moderate levels of Hg. According to Figure 8b, heavy metal Hg reached significant contamination in 7% of the soil samples in the study area, which indicates the need to pay attention to Hg contamination in agricultural soils in the region.

#### 3.5.5. Potential Ecological Risk Index

The potential hazard risk index method, proposed by the Swedish scholar Hakanson (1980) [24], was an index to investigate the extent of heavy metal pollution using the biotoxicity coefficient of heavy metals and the ratio of heavy metal content in sediments to the geological background value [45]. This method not only considers the relationship between the content of heavy metals and the background value but also considers the biological toxicity of heavy metals, which can provide a reference for people’s health and is a more frequently used method to evaluate heavy metal pollution [46,47]. The E_r_ ranged from 2.92 to 16.89 for As, 8.45 to 74.91 for Cd, 3.20 to 9.11 for Co, 0.64 to 3.62 for Cr, 0.83 to 19.88 for Cu, 12.83 to 476.17 for Hg, 0.60 to 1.82 for Mn, 2.24 to 8.87 for Ni, 2.49 to 14.24 for Pb, 0.59 to 2.26 for Zn (Table 6). The average values of E_r_ for As, Cd, Co, Cr, Cu, Hg, Mn, Ni, Pb, and Zn were 9.29, 38.88, 5.98, 2.03, 8.38, 102.60, 1.20, 5.52, 6.95, and 1.36, respectively. According to the found concentrations and classification criteria, the ecological risk of As, Cd, Co, Cr, Cu, Mn, Ni, Pb and Zn was low and that of Hg was high in the agricultural soil samples collected in the study area. The result of the comprehensive potential ecological risk index showed that the combined ecological risks in the study area were moderate.

As shown in Table 7, the As, Co, Cr, Cu, Mn, Ni, Pb, and Zn were low ecological risk in all samples. The percentage of moderate ecological risk of Cd was 47%. Furthermore, 33%, 11%, and 4% of Hg in the study area samples reached considerable ecological risk, high ecological risk, and extreme ecological risk. The result of the comprehensive potential ecological risk index showed that 42% of the samples were low ecological risk, 50% of samples were moderate ecological risk and 8% of samples were a considerable ecological risk. This indicated that the contamination of Cd and Hg was more serious in the study area, and the overall heavy metal contamination was moderate.

**Figure 9:**
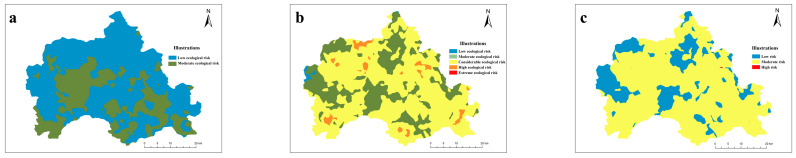
(**a**) Map of ecological risk distribution in the study area (Cd), (**b**) Map of ecological risk distribution in the study area (Hg), and (**c**) Integrated potential ecological risk map of the study area.

Using ordinary Kriging to map the pollution of major priority heavy metals in the study area. The Cd moderate ecological risk areas in the study area showed a blocky distribution, mainly concentrated in the central, southwestern, and southeastern parts (Figure 9a). The areas of severe Hg high ecological risk in the study area were distributed in a point pattern. Overall, there were significantly more high-value areas around than in the center (Figure 9b). The integrated pollutant mapping results showed that the risk of heavy metal pollution in the study area was mostly moderate, except for the western and central parts of the region (Figure 9c).

### 3.6. Comparison of Estimation Methods for Heavy Metal Pollution

Based on the previous analysis, the pollutant levels were calculated for the four methods. The results showed no significant difference in the results of the four methods (Table 8 and Table 9). Similar contamination levels were obtained for the single factor index and the enrichment index. The contamination levels obtained using the geo-accumulation index were equivalent to those received using the potential ecological risk index.

Table 10 shows the ranking of heavy metal contamination obtained by various methods. The single factor index, the Nemerow composite index, the enrichment index, and the geo-accumulation index had the same results. However, the potential ecological risk index had different results from the other three methods.

## 4. Discussion

Our results showed that the coefficient of variation of heavy metals was larger in typical mountainous, hilly areas. Studies have shown that heavy metals are highly variable and susceptible to human activities [48,49]. The strong variation of Hg in the study area suggests that there is a large variation in the spatial distribution of heavy metal Hg in the region, and the content difference between sampling points is very large [50]. We also found that the soil organic matter content in the study area was only in the middle of the country. This may be related to the fact that the study area belongs to a mountainous, hilly area. Mountain soils are usually less fertile than lowlands, and steep slopes accelerate erosion, with broad effects on larger downstream ecosystems [51,52]. The results of the correlation analysis indicated that soil organic matter and pH were important indicators of the heavy metal content in the soil. [53,54,55]. All heavy metal elements are positively correlated indicating that heavy metal contamination of agricultural soils in the region may have the same source [23,56].

The result of PCA showed that the first principal component (PC1) was mainly composed of Ni, Co, Cr, and Mn. The common information of the three heavy metal elements, Cd, Pb and Hg, is reflected in the second principal component. In a related study by scholars, Co and Mn were assigned to the same principal component and analyzed to conclude their origin from weathered rocks [57]. The aggregation of two elements, Cr and Ni, in the soil is also highly correlated with the soil-forming parent material. Cr and Ni are both Fe-group elements with ferrophilic properties and generally have a high correlation in the soil [58]. The rocks in the study area were deposited into the soil through weathering resulting in high concentrations of some heavy metals [59]. Therefore, it can be assumed that the elements in PC1 are mostly of natural origin in the soil. The main sources of the three elements Cd, Pb, and Zn are industrial waste emissions [60]. It was shown in related studies that road dust contains relatively high levels of Cr and Pb (EF > 2) from traffic exhaust and non-exhaust emissions, while moss plants can accumulate high concentrations of Pb and Zn from the deposition of traffic emissions [61]. Elemental Hg aggregates are mainly emitted to the atmosphere through coal combustion, power plants and coking plants and enter the soil mainly through dry atmospheric deposition and wet deposition [58,62]. This suggested that the elements in PC2 might be mainly derived from industrial and agricultural pollution.

The results of geostatistical analysis and mapping found that the high-value areas of heavy metal pollution are mostly found in the plain areas. Human activities in mountainous and hilly areas are mostly concentrated in plain areas, and pollution from human activities is also an important cause of heavy metal enrichment [63,64]. The concentration of industrial and agricultural industries is an important reason for the concentration of heavy metal pollution in the plain areas of the study area.

Both quantitative and qualitative food security is an important topic for global sustainable development [65,66]. Contamination of the food chain with heavy metals is considered one of the major environmental pathways of human exposure to metals leading to potential health risks [67]. Heavy metals can be persistent and cumulative in soil, increasing the toxicity when combined with inorganic and organic matter [68]. Soil heavy metals can accumulate in the food chain, followed by food consumption into the human body. Heavy metals can also accumulate in the human body via dermal contact absorption, direct ingestion and inhalation. Drinking of water and inhalation of soil particles have been identified as the major pathway for human exposure to toxic metals [11,69].

Adverse effects of heavy metals and metal compounds on soil organisms through microbial processes and soil-microbial interactions [70]. Most heavy metals can accumulate in crops grown on soils contaminated with heavy metals and then be transferred to humans through the food chain. A number of methods have been developed to assess the extent of soil contamination and its potential risk to human health, and a number of soil-plant transfer indices have been used to assess the environmental safety risk of heavy metals in soil [67].

The heavy metal pollution index study showed the most serious Hg pollution in the region, and the results of various methods did not differ significantly; only the results of the ecological risk index analysis were slightly different from others. Such differences result from ecological risk factors calculated from different toxicological response factors [71]. Furthermore, Cd and Hg are long known for their considerable environmental risk [68]. Relevant studies have shown that due to the excessive heavy metals in the soil of some areas where rice is grown, the adsorption capacity of rice for cadmium pollution is also higher than that of other crops, resulting in the production of rice in these areas with a serious excess of cadmium content. Thus, the population’s daily intake leads to diseases in many villagers [71]. At the same time, a study surfaced, in the Guizhou area, adults through rice daily intake of mercury up to 49 micrograms.

The limitation of this study was that we have not been able to obtain data on food samples in the studied area to investigate further the heavy metal soil–crop–human relationship. We are currently obtaining this part of the data, and we will further refine this part of the study in the future.

## 5. Conclusions

In farmland soils, for 10 heavy elements (As, Cd, Co, Cr, Cu, Hg, Mn, Ni, Pb and Zn), with mean concentrations of 10.07, 0.14, 13.64, 64.81, 35.21, 0.05, 657.01, 30.9, 26.42 and 81.8, respectively. Results showed lower average levels of heavy metals than the risk screening values of the Chinese national soil environmental quality standards (GB 15618-2018). However, the average content of heavy metals (Cd, Co, Cr, Cu, Hg, Mn, Ni, Pb, and Zn) exceeds the Shandong background values by factors of 1.27, 1.20, 1.01, 1.68, 2.78, 1.20, 1.10, 1.39, and 1.36, respectively.

As the results of the correlation analysis suggested, the heavy metals in the study area may have the same source and that soil pH and organic matter content may influence the accumulation of some heavy metals in the soil. According to the results of the principal component analysis, the sources of heavy metals in the soils of the study area can be divided into two classes, the first category contains seven heavy metals, Ni, Co, Cr, and Mn, and the second category contains three heavy metals Cd, Pb, and Hg.

There were results of single factor index, Nemerow index, enrichment index and geoaccumulation index, which showed that the decreasing order of heavy metal pollution was Hg > Cu > Pb > Zn > Cd > Mn > Co > Ni > Cr > As. According to the results of the potential ecological risk index, the decreasing order of heavy metal pollution is Hg > Cd > As > Cu > Pb > Co > Ni > Co > Zn > Mn. The most serious was Hg pollution in the study area. The integrated pollutant mapping results showed that the risk of heavy metal pollution in the study area was mostly moderate, except for the western and central parts of the region.

We found that heavy metal contamination of agricultural soils has a significant impact on crop growth and human dietary intake, and the exposure can lead to the accumulation of heavy metals in the human body, which in turn affects human health. Therefore, this study exhibited that an enhanced and detailed analysis of the characteristics and sources of heavy metal contaminants in farmland soils can facilitate the monitoring of heavy metal pollution at the primary stage of the food chain and assess the risk of the presence of heavy metal pollution in food, thus, improving the health of the residents.

In our future work, we will continue to study the hazards of potentially toxic elements in farmland soils to humans, clarify the potential transfer model between farmland and humans, and refine the assessment of potential toxic elements in soils to human health risks.

## Figures and Tables

**Table 1:** The limits of soil indicators.

Indicators	Units	LOQ	LOD
As	mg/kg	4	1
Cd	mg/kg	0.08	0.02
Co	mg/kg	1.6	0.4
Cr	mg/kg	16	4
Cu	mg/kg	4	1
Hg	mg/kg	0.002	0.0005
Mn	mg/kg	32	8
Ni	mg/kg	8	2
Pb	mg/kg	4	1
Zn	mg/kg	10	2.5
SOM	%	0.4	0.1

Abbreviations: As—Arsenic; Cd—Cadmium; Co—Cobalt; Cr—Chromium; Cu—Cuprum; Hg—Hydrargyrum; Mn—Manganese; Ni—Nickel; Pb—Plumbum; Zn—Zinc; pH—Potential of hydrogen; SOM—soil organic matter; LOQ—Limits of Quantification; LOD—Limits of Detection. Note: The limit of quantification was calculated as four times the limit of detection.

**Table 2:** Descriptive statistics of heavy metal concentrations, pH and SOM in farmland soil samples (*n* = 3060).

	Min	Max	Mean	SD	CV	S	K	B-Values
As	3.06	17.73	10.07	3.23	0.32	−0.02	−0.43	10.5
Cd	0.03	0.27	0.14	0.05	0.35	0.28	−0.29	0.11
Co	7.29	20.76	13.64	2.91	0.21	0.12	−0.39	11.4
Cr	20.57	115.80	64.81	16.11	0.25	−0.01	0.60	64
Cu	3.48	83.48	35.21	14.57	0.41	0.90	0.72	21
Hg	0.01	0.21	0.05	0.04	0.77	2.12	5.07	0.018
Mn	330.40	996.50	657.01	147.63	0.22	−0.09	−0.51	547
Ni	12.53	49.69	30.90	7.17	0.23	−0.42	−0.11	28
Pb	9.45	54.12	26.42	6.05	0.23	1.40	3.48	19
Zn	35.14	135.30	81.80	17.82	0.22	0.19	0.09	60
SOM	0.24	7.34	1.89	0.85	0.45	1.46	4.08	-
pH	4.54	8.79	7.36	0.93	0.13	−0.87	−0.21	-

Abbreviations: St.D—Standard Deviation; CV—Coefficient of Variation; K—Kurtosis; S—Skewness.

**Table 3:** Correlation analysis of soil heavy metals with pH and SOM.

	As	Cd	Co	Cr	Cu	Hg	Mn	Ni	Pb	Zn
pH	0.067 **	0.051 **	0.077 **	0.066 **	0.063 **	−0.026	0.081 **	0.130 **	−0.032	0.112 **
SOM	0.062 **	0.010	0.025	0.039 *	0.032	−0.029	0.016	0.072 **	−0.019	0.046 *

Note: **. Significant correlation at the 0.01 level (two-tailed). *. Significant correlation at the 0.05 level (two-tailed).

**Table 4:** Factors matrix of metal elements in soils.

PC	Factor Matrix										C (%)	C-c (%)
	As	Cd	Co	Cr	Cu	Hg	Mn	Ni	Pb	Zn		
PC1			0.817	0.795			0.725	0.838			35.70	35.70
PC2		0.691				0.635			0.689		18.58	54.28

Note: Extraction Method: Principal Component Analysis. Rotation Method: Varimax with Kaiser Normalization. Only list factor matrix greater than 0.6. Abbreviations: C—Contribution; C-c—Cumulative contribution.

**Table 5:** Nemerow composite index.

Element	Value	Risk
As	1.37	Slight contamination
Cd	1.99	Slight contamination
Co	1.54	Slight contamination
Cr	1.47	Slight contamination
Cu	3.05	Severe contamination
Hg	8.61	Severe contamination
Mn	1.54	Slight contamination
Ni	1.48	Slight contamination
Pb	2.24	Moderate contamination
Zn	1.86	Slight contamination

**Table 6:** Description of the results of the potential ecological risk index.

	Min	Max	Mean	St.D
As	2.92	16.89	9.59	3.07
Cd	8.45	74.91	38.88	13.62
Co	3.20	9.11	5.98	1.27
Cr	0.64	3.62	2.03	0.50
Cu	0.83	19.88	8.38	3.47
Hg	12.83	476.17	102.60	79.36
Mn	0.60	1.82	1.20	0.27
Ni	2.24	8.87	5.52	1.28
Pb	2.49	14.24	6.95	1.59
Zn	0.59	2.26	1.36	0.30
RI	51.60	573.64	182.50	86.18

Abbreviations: RI—the comprehensive potential ecological risk index.

**Table 7:** The result of the percentage of E_r_ and RI grades.

Element	Low Ecological Risk	Moderate Ecological Risk	Considerable Ecological Risk	High Ecological Risk	Extreme Ecological Risk
As	3060 (100%)	0 (0%)	0 (0%)	0 (0%)	0 (0%)
Cd	1622 (53%)	1438 (47%)	0 (0%)	0 (0%)	0 (0%)
Co	3060 (100%)	0 (0%)	0 (0%)	0 (0%)	0 (0%)
Cr	3060 (100%)	0 (0%)	0 (0%)	0 (0%)	0 (0%)
Cu	3060 (100%)	0 (0%)	0 (0%)	0 (0%)	0 (0%)
Hg	384 (13%)	1211 (40%)	1019 (33%)	334 (11%)	112 (4%)
Mn	3060 (100%)	0 (0%)	0 (0%)	0 (0%)	0 (0%)
Ni	3060 (100%)	0 (0%)	0 (0%)	0 (0%)	0 (0%)
Pb	3060 (100%)	0 (0%)	0 (0%)	0 (0%)	0 (0%)
Zn	3060 (100%)	0 (0%)	0 (0%)	0 (0%)	0 (0%)
RI	1272 (42%)	1517 (50%)	271 (8%)	0 (0%)	0 (0%)

**Table 8:** Pollution grades of each element with different assessment methods.

Assessment	As	Cd	Co	Cr	Cu	Hg	Mn	Ni	Pb	Zn
P_i_	I	II	II	II	II	III	II	II	II	II
P_n_	III	III	III	III	IV	V	III	III	IV	III
*I_geo_*	I	I	I	I	II	II	I	I	I	I
EF	I	II	II	I	II	III	II	I	II	II
E_r_	I	I	I	I	I	III	I	I	I	I

**Table 9:** Statistics of heavy metals in farmland soils in the study area.

Assessment	Statistics	As	Cd	Co	Cr	Cu	Hg	Mn	Ni	Pb	Zn
	Min	0.29	0.28	0.64	0.32	0.17	0.32	0.6	0.45	0.5	0.59
P_i_	Max	1.69	2.5	1.82	1.81	3.98	11.9	1.82	1.77	2.85	2.26
	Mean	0.96	1.3	1.2	1.01	1.68	2.57	1.2	1.1	1.39	1.36
	Min	−2.36	−2.41	−1.23	−2.22	−3.18	−2.23	−1.31	−1.75	−1.59	−1.36
*I_geo_*	Max	0.17	0.74	0.28	0.27	1.41	2.99	0.28	0.24	0.93	0.59
	Mean	−0.73	−0.31	−0.36	−0.62	0.04	0.45	−0.36	−0.49	−0.14	−0.17
	Min	0.11	0.11	0.36	0.2	0.24	0.12	0.26	0.33	0.16	0.41
EF	Max	4.79	5.4	5.61	4.87	10.51	31.88	5.87	5.4	8.48	5.45
	Mean	0.83	1.14	1.06	0.88	1.42	2.27	1.06	0.97	1.28	1.2
	Min	2.92	8.45	3.2	0.64	0.83	12.83	0.6	2.24	2.49	0.59
E_r_	Max	16.89	74.91	9.11	3.62	19.88	476.17	1.82	8.87	14.24	2.26
	Mean	9.59	38.88	5.98	2.03	8.38	102.6	1.2	5.52	6.95	1.36

**Table 10:** Decreasing order of heavy metal pollution.

Assessment	Order
P_i_	Hg > Cu > Pb > Zn > Cd > Mn = Co > Ni > Cr > As
P_n_	Hg > Cu > Pb > Zn > Cd > Mn > Co > Ni > Cr > As
*I_geo_*	Hg > Cu > Pb > Zn > Cd > Mn > Co > Ni > Cr > As
EF	Hg > Cu > Pb > Zn > Cd > Mn > Co > Ni > Cr > As
E_r_	Hg > Cd > As > Cu > Pb > Co > Ni > Co > Zn > Mn

## Data Availability

The data presented in this study are available on request from the corresponding author.

## Supplementary Materials

The following supporting information can be downloaded at: https://www.mdpi.com/article/10.3390/foods12030681/s1, Table S1: Classification of pollution degrees.
