# Supplementary material for: Pollution Characteristics, Spatial Distribution, and Evaluation of Heavy Metal(loid)s in Farmland Soils in a Typical Mountainous Hilly Area in China
Source: Foods. 2023 Feb 3;12(3):681. doi: 10.3390/foods12030681 (PMC9913997; doi:10.3390/foods12030681)
Supplement: Supplementary file 1 [file foods-12-00681-s001.zip › foods-2031682-supplementary.pdf]

Pollution characteristics, spatial distribution, and evaluation of heavy metal(loid)s in farmland soils in a typical mountainous hilly area of China

Guohui Shen[1], Xin Ru[1], Yanting Gu[1], Wei Liu[2], Kunzhen Wang[1], Baiyi Li[1], Yanzhi Guo[3,*] and Juan Han[1,*]

[1] Institute of Food and Nutrition Development, Ministry of Agriculture and Rural Affairs, Beijing 100081, China

[2] North China Geological Prospecting and Ecological Resources Monitoring Center (Hebei), Chengde 067000, China

[3] Department of Research Management, Chinese Academy of Agricultural Sciences, Beijing 100081, China

* Correspondence: guoyanzhi@caas.cn (Y.G.); hanjuan@caas.cn (J.H.); Tel.: +86-10-8210-6427; Fax: +86-10-8210-5184

Supplement

Table S1. Classification of pollution degrees

Class	P_i	Pollution degree	P_N	Pollution degree	I_{geo}	Pollution degree	EF_i	Pollution degree	E_r	Pollution degree	RI	Pollution degree
Ⅰ	≤1	Safe	≤0.7	Safe	≤0	Unpolluted	<1	No pollution	<40	Low ecological risk	<150	Low risk
Ⅱ	(1,2]	Warning Threshold	(0.7,1]	Warning Threshold	（0,1]	Unpolluted to moderately	(1,2)	Minimal pollution	[40,80)	Moderate ecological risk	[150,300)	Moderate risk
Ⅲ	(2,3]	Slight Contamination	(1,2]	Slight Contamination	（1,2]	Moderately	(2,5)	Moderate pollution	[80,160)	Considerable ecological risk	[300,600)	High risk
Ⅳ	(3,5]	Moderate Contamination	(2,3]	Moderate Contamination	（2,3]	Moderately to strongly	(5,20)	Significant pollution	[160,320)	High ecological risk	≥600	Extreme risk
Ⅴ	>5	Severe Contamination	>3	Severe Contamination	（3,4]	Strongly	(20,40)	very Strong pollution	≥320	Extreme ecological risk		
Ⅵ					(4,5]	Strongly to extremely	>40	Extreme pollution				
Ⅶ					>5	Extremely						
